# Older People’s Voices Matter!: Possibilities and Limitations for Providing Dignified Palliative Care with Wearable Devices

**DOI:** 10.1177/23333936251375182

**Published:** 2025-09-27

**Authors:** Rada Sandic Spaho, Lisbeth Uhrenfeldt, Theofanis Fotis, Jorunn Bjerkan, Ingjerd Gåre Kymre

**Affiliations:** 1 Nord University, Bodo, Norway; 2 Lillebaelt Hospital, Kolding, Denmark; 3 Southern Danish University, Odense, Denmark; 4 University of Brighton, Eastbourne, United Kingdom; 5 Nord University, Levanger, Norway

**Keywords:** aged, dignity, patient attitudes, digital health, digital monitoring, pain management, interprofessional collaboration, peer-support, Norway

## Abstract

This study aimed to gain insight into the phenomenon of how wearable devices affect the sense of dignity of older patients (above 65 years of age) in palliative care settings using remote monitoring health services. Five interviews were carried out using reflective lifeworld research approach. A phenomenological analysis showed that the essence of older adults’ sense of dignity in palliative care is vulnerable and, therefore, at stake when using wearable devices for remote monitoring. This essence is reflected in the following constituents: a simultaneous sense of regained and diminished quality of life; mixed feelings ranging from strengthened self-identity and independence to vulnerability and dependence; shifting care responsibilities within the context of life’s finitude; and uncertainty about being valued, seen, and empowered while navigating relational expectations. The phenomenon was illuminated through the lens of various forms of dignity, offering insight into how it is experienced and challenged. These findings underscore the importance of recognising and addressing threats to dignity as digital health technologies continue to evolve in palliative care settings. Interprofessional collaboration among various stakeholders, including patients as active informants and participants, is necessary for designing future dignified remote care using wearable devices.

## Introduction

Having a vulnerable or frail body, depending on others, and being unable to maintain one’s own standards may affect older patients’ sense of dignity in palliative care settings. Older people with multiple comorbidities and cancer patients are especially sensitive and may be easily affected (Galvin & Todres, 2013, 2015; Söderman et al., 2020). Patients receiving palliative care have limited lifespans, and especially when coupled with comorbidities, may be in a vulnerable position as they depend on care from a variety of healthcare services and professionals (Fernando & Hughes, 2019; Melby & Håland, 2021; Mertens et al., 2021). Palliative care is a complex type of care that provides symptom management and alleviates suffering in life-threatening illnesses while at the same time improving the patient’s quality of life by supporting the psychological, spiritual, and social needs of both patients and family members (Teoli et al., 2023; WHO, 2020).

Considering that dignity and palliative care are human rights, maintaining a patient’s dignity is very important in palliative care (Östlund et al., 2019; Söderman et al., 2020). Maintaining someone’s dignity can be interpreted as valuing them despite their vulnerability (Galvin & Todres, 2015) through empowerment, care, compassion, empathy, kindness, and respect (Söderman et al., 2020). The findings from Åberg et al. (2020) study with older people living with long-term multiple comorbidities highlighted that they see the ability to make their own decisions about their medications, residences, and activities as very important. Taking away this ability creates feelings of diminishment and dependence and denies the patients’ rights to make their own choices (Åberg et al., 2020). Patients in palliative care feel that their dignity is supported when they are heard and given a chance to decide about their treatments, their needs, and their quality of life and when they feel empowered and hopeful that they will be able to do things they used to do before their illness, while still experiencing autonomy over their lives (Bovero et al., 2024; Östlund et al., 2019).

However, 86% of people in need of palliative care are being overlooked in receiving the care they need because of ongoing worldwide obstacles, including inadequate access to care, medications, resources, insufficient training of healthcare professionals, and lack of or inadequate implementation of national policies (Söderman et al., 2020; WHO, 2020). The digitalisation of health systems is seen as a way to address the increased challenges and demands in providing healthcare (European Commission, 2018b), to the global growth of the population who are ageing with multiple comorbidities (Siemens et al., 2020; United Nations, 2019), and the increasing shortage of millions of healthcare professionals (Liu et al., 2017; WHO, n.d.).

Digital health comprises eHealth, mHealth, wearable devices, the Internet of Things, telemedicine, and telehealth (WHO, 2018, 2022). It can reduce hospital readmissions and treatment costs while improving healthcare professionals’ efficiency (European Commission, 2018a; Maguraushe & Ndlovu, 2024; Nene et al., 2023). Specifically, telemedicine, wearable devices, and remote home monitoring come under healthcare services that provide care at a patient’s home (Ovid Technologies Inc., 1993). By 2030, the world will use 50 billion different Internet of Things products. Their emergence in healthcare allows many possibilities, especially with remote home monitoring (Rejeb et al., 2023).

Remote home monitoring can be offered via various health services, such as enabling patients to use wearable devices, read measurements, communicate with healthcare professionals via mobile applications, and adjust their treatments accordingly. It can also involve video or telephone consultations, messaging between patients and healthcare professionals, and/or patients’ reporting their own measurements (Haase et al., 2020; Helsedirektoratet [Ministry of Health Department], 2021; Schmalz et al., 2020; Walton et al., 2022). Wearable devices are usually connected to remote or digital home monitoring departments, which provide remote care delivery while the patient is at home. Wearable devices are defined as wearable technology on the patient’s body (Cardona et al., 2021; Koszalinski et al., 2019). These devices may have sensors that send patients’ measurements (e.g., heart rate, sleep patterns, temperature) digitally via router and/or mobile application to healthcare providers. They can be GPS trackers, activity trackers, gait monitors for preventing falls, or devices that have only one function, such as safety alarms to activate emergency calls (Cannon, 2018; Cardona et al., 2021; Koszalinski et al., 2019; Patel et al., 2015; Sandic Spaho et al., 2023). Such technology can offer patient-centred care at the patient’s residence without the need for travelling, enabling necessary care when needed, while reducing costs and hospital readmissions (Cannon, 2018; Cardona et al., 2021; Sandic Spaho et al., 2023; Walton et al., 2022). Including patients as active participants in the healthcare system allows them to become more involved in their own symptom and treatment management (Walton et al., 2022).

However, patients may experience this kind of healthcare in different ways. While they may feel empowered to manage their own symptoms, they may also feel pressured and unprepared for such responsibility (Schmalz et al., 2020; Walton et al., 2022). So far, studies using different models of remote home care have shown that patients value text-based communication with healthcare professionals (HCPs) and the possibility of having insight into their own data as necessary, as this enables better and earlier symptom management and increases the patient’s confidence and feelings of safety (Helsedirektoratet [Ministry of Health Department], 2021; LeBaron et al., 2022; Schmalz et al., 2020). Remote home monitoring with wearable devices may offer possibilities such as patient autonomy and patient-centred care at the patient’s residence, while maintaining the feasibility of this type of care. Nevertheless, the scientific literature has not discussed its impact on patients’ sense of dignity. The literature on patients’ lived experiences of using wearable devices, especially in palliative care contexts, and for the population older than 65, is sparse (Nene et al., 2023; Pavic, Klaas, Theile, Kraft, Tröster, Blum, & Guckenberger, 2020; Pavic, Klaas, Theile, Kraft, Tröster, & Guckenberger, 2020; Sandic Spaho et al., 2023).

The aim of this study is to gain insight into the phenomenon of how wearable devices affect the sense of dignity of older patients (above 65 years of age) in palliative care settings using remote monitoring health services. Increasing our knowledge about patients’ lived experiences with wearable devices could enhance our understanding of the phenomenon and the ability to provide personalised, dignified palliative care.

## Method

To gain insight into the phenomenon of how wearable devices affect the sense of dignity of older patients in palliative care settings using remote monitoring health services, Reflective Lifeworld research was applied as an approach (Dahlberg et al., 2008). This approach enables the description of a phenomenon and its deeper meanings (Dahlberg et al., 2008) and has its roots in the phenomenology of the lifeworld. A phenomenological analysis was conducted to describe the essence and constituents of the phenomenon. Phenomenology research phenomenon, things, or experiences as they show themselves. The phenomenon and its constituents, derived from the deeper meanings behind participants’ words, rather than individual experiences, are at the centre of phenomenology. According to the philosopher Maurice Merleau-Ponty, we experience the world through our bodies (Dahlberg et al., 2008). For this reason, it can be assumed that wearable devices in the context of palliative care affect the lived experience of patients older than 65 through their bodies. Reflective Lifeworld research proposes bridling, in which the researcher strives for openness against her/his preunderstanding. Bridling requires openness for infinite possible understandings of the phenomenon, so that researchers “do not understand too quickly, too carelessly or slovenly, or in other words, they do not make definite what is indefinite” (Dahlberg et al., 2008, p. 130). Researchers’ striving for bridling openness positioning started from planning a research and interview guide, followed by data gathering through interviews, in a way that patients were allowed and encouraged to tell as much as possible and as much as they wanted to about the topic.

### Participants and Data Collection

The inclusion criteria specified that participants should be patients older than 65 who used a wearable device and received palliative care. Participants were recruited through different healthcare institutions and their gatekeepers. The recruitment period, which spanned from October 2021 to June 2023, was limited by the COVID-19 pandemic and the project’s duration, which concluded in June 2023. The process was lengthy and limited by restricted access to appointments during the COVID-19 pandemic.

Fourteen patients were recruited, but before the interviews were held, three patients died, four patients’ health conditions worsened, and two patients withdrew before the interview, leaving a total of five interviewed patients. All patients were contacted and interviewed 1 to 3 days after their contact details were obtained, at a time and place of their choice. One patient gave consent, but in the meantime got worse and died before he could be interviewed.

A semi-structured interview with open and reflective attitude was enabled by asking questions, directing to the phenomena of patients’ sense of dignity, asking additional follow-up questions, as well as asking patients to give examples and to reflect on what they said, and what it means to them, in order to gain rich data about the phenomenon. Patients’ descriptions of lived experiences should be comprehensive, specific, and as explicit as possible (Dahlberg et al., 2008).

The five interviewed patients were three women and two men, aged 67 to 93 years, with a mean age of 78.6 years. One patient was interviewed at home; two were interviewed in their assisted living facilities, which had a small number of HCPs available, only during the day shift, for all the residents; one was interviewed at the short-stay municipal hospital; and one was interviewed at the apartment provided by the specialist hospital immediately after hospital discharge. All participants received care at the primary care level, organised by the municipality, which included short-stay municipal hospitals, home care services, family physicians, and limited specialist care services (La Rocca & Hoholm, 2017; Spaho et al., 2024). Participants lived in several inland and coastal cities, which varied in size from 3,000 citizens to more than 50,000 citizens (considered as a big city). They were associated with two regional specialist hospitals, organised by regional authorities, which, for some patients, were located between 150 and 500 km away from their place of residence. Home care services were available in each city. Remote home monitoring services were located in major cities, organised differently in each city, but in some cases were part of home care services.

Details of the participants’ characteristics and the types of wearable devices they were using are presented in Table 1.

**Table 1.** Participants’ Characteristics and the Types of Wearable Devices They Used.

Patient number	Age	Sex	Marital status	Place	Receiving home care	Blood pressure device	Weight scale	Safety alarm	Pain pump	Medication dispenser
1	72	M	Married	ALF	x	x	x	x		X
2	67	F	Married	Hospital				x	x	
3	93	F	Widow	ALF	x			x		
4	80	F	Married	Home	x			x		X
5	81	M	Married	ALF	x				x	

*Note*. M = male; F = female; ALF = assisted living facility.

The first (RSS) and last (IGK) authors developed a semi-structured interview guide allowing for flexibility and dialogue. The questions were adapted to match the types of wearable devices and remote care services used by participants. A digital monitoring department provided remote care for blood pressure and weight scales, monitoring daily measurements. Home care services provided pain pumps. Safety alarms were provided by remote healthcare services, which sometimes cooperated with home care. The first author conducted all the interviews, in two cases assisted by a fellow researcher who took notes. The interviews were held between November 2022 and June 2023. Each interview lasted approximately 60 min and was held at a time and place chosen by the participant. In lifeworld research, there is no saturation of data, as ontologically and epistemologically, meanings are never-ending and constantly expanding. Accounting for variation in data collection, such as differences in age and culture, is essential to ensure richness and relevance in the findings (Dahlberg et al., 2008). In this study, diversity was addressed through the inclusion of participants from both sexes and varied residential contexts, as well as the use of different types of wearable devices. The participants valued that the first author came to their residence, taking the time to reflect with them, asking for examples, and following up with additional questions. In one case, a pause was made during the interview when the patient wanted to change clothes to feel more dignified, which deepened reflections on how the patient experiences dignity. Nonetheless, participants reflected on the usefulness of their opinions due to their age, which is elaborated upon in the results section.

### Analysis

After data gathering, the same open approach was applied in the further analysis of the material. In this study, bridling is used to let the phenomenon of how wearable devices affect the sense of dignity of older patients in palliative care settings using remote monitoring health services shows itself and its constituents. By bridling researcher is striving for openness to the myriad meanings, not just the immediate ones. Researchers search for profound meanings behind patients’ words while shifting between objectivity and subjectivity, taking the time to reflect on the data material during analysis.

Phenomenological analysis was conducted with the aim of providing a description of the phenomenon’s essence and its constituents, as well as how wearable devices affect the sense of dignity in older patients (above 65 years of age) in palliative care settings using remote monitoring health services. Since the Reflective lifeworld research approach implies “bridling openness” (Dahlberg et al., 2008) the interview data were read several times to ensure a thorough understanding of the whole picture. A scientific attitude requires openness and reflection, which helps with forming different meaning units. Similar units were temporarily grouped in clusters. The essential meanings elucidating the phenomenon were constantly seen in the parts and in the whole. Clusters were connected to describe the phenomenon and its constituents. The phenomenon was described in terms of the essence, assembled from the constituents. As Dahlberg et al. (2008, p. 247) say: “*essences are their phenomena, the phenomena are their essences*.” All the constituents together and their essences described the phenomenon as it showed itself as that particular phenomenon.

Validity, objectivity, and quality of the study were ensured by bridling, separating the researchers’ previous knowledge by striving for being open and reflective throughout the data gathering, data analysis, transparency, and going back and being sensitive to “things themselves” and the phenomenon. Numerous hours were spent in reflections and discussions between the first (RSS) and last (IGK) authors about the meanings and the phenomenon (Dahlberg et al., 2008).

## Results

The phenomenon of older patients’ sense of dignity in palliative care, as experienced through the use of wearable devices, was explored through phenomenological analysis. The analysis showed that the essence of older adults’ sense of dignity in palliative care is vulnerable and, therefore, at stake when using wearable devices for remote monitoring. This experience is marked by ambivalence, expressed through mixed emotions and conflicting perceptions of whether the care is dignified. These tensions are further illustrated in the following constituents:

- Simultaneous sense of regained and diminished quality of life.
- Mixed feelings ranging from strengthened self-identity and independence to vulnerability and dependence.
- Shifting care responsibilities within the context of life’s finitude.
- Uncertainty about being valued, seen, and empowered while navigating relational expectations.

### Simultaneous Sense of Regained and Diminished Quality of Life

Wearable devices and remote monitoring introduce a new dimension to quality of life, supporting a sense of dignity by enhancing safety and enabling timely assistance. They allow individuals greater autonomy, such as the freedom to travel, walk without pain, and better understand their health, measurements, and medications, thereby reinforcing a sense of control and self-respect in care. Ambivalence emerges from the feeling that one aspect of quality of life is regained, when another is diminished. Senses of dignity fluctuate from regained life possibilities and control to feeling vulnerable, frail, and restricted in noting diminished ability to do things as usual.

A patient wearing a pain pump describes the possibility of a new quality of life as follows: “*If I don’t press the button for an extra dose in time, my whole world falls apart in pain. But the pain pump adds to my quality of life, relieving all the pain and not just cancer pain. It is giving me not just a feeling of safety but a whole new world of possibilities*” (P2). Patients appreciate being in control of getting care when they need it by using wearable devices.

In contrast, a loss of quality of life is expressed in this way: “*The pain pump made me pain-free, and that’s good. But now, because of morphine, I can’t drive anymore. I feel depressed because I’ve lost the ability to drive, and driving was a huge part of my life that I enjoyed*” (P5). Having to give up a significant element in what had made life enjoyable despite the disease created a feeling of loss in these patients’ quality of life. It caused mixed feelings towards what wearable devices offer, including a sense of compromised identity and feeling vulnerable and limited.

### Mixed Feelings Ranging from Strengthened Self-Identity and Independence to Vulnerability and Dependence

Wearable devices represent a renewed sense of autonomy, feelings of safety, and opportunities to go on with their lives without depending on others. Maintaining self-identity is appreciated by the possibility to ask for help, when and how much they want. However, a question arises concerning the autonomy they had gained when reflecting on their health deterioration and whether wearable devices will continue to be helpful. The sense of dignity oscillated between feeling worthy, having a deep sense of self-identity, independence, and self-ability on the one hand, and feeling vulnerable and constrained, both bodily and by the devices, on the other.

A sense of enhanced autonomy is expressed through feelings of responsibility, advantage, and empowerment gained by using wearable devices, including the ability to maintain life routines and reduced dependence on healthcare professionals. Autonomy is also expressed through renewed satisfaction with oneself, despite diseases, and through the ability to receive care at home or in a facility that enables independence.

Digital monitoring gives insight into own measurements. As an example, one patient felt self-contained, independent, and in charge of their own health, knowing and trusting that the HCPs monitoring team would react if measurements went outside the normal range. Similarly, medication dispensers help patients stick to their medication intake routines by alerting them to take their medications at the recommended times at home. Pain pumps permit them to adjust their own morphine doses as needed to stay pain-free and self-sufficient. Safety alarms allow treatments to be adjusted and disease complications to be resolved without requiring a trip to a healthcare setting, also enabling immediate help in case of a fall, severe health deterioration, or even when simply not feeling well. In such a case, they can press a button to initiate contact with healthcare services.

I have had enough [experience with the safety alarm] with cancer, fractures, treatments. . . I fell a few times in the bathroom, and paramedics came and took me to the hospital [after I activated the safety alarm]. I feel safe when I see my measurements, knowing I am [digitally] monitored and can call and get help. (P1)

However, not everyone seems to accept wearable devices to the same extent. An older patient said that the residents of their assisted living facility felt diminished and downgraded when safety alarms replaced the HCPs’ night shifts. They felt their opinions and protests had not been considered in the decision-making process. Each patient resides in their own apartment, which features one bedroom. The speakerphones for the safety alarms were installed in the hallways of the patients’ apartments, and not in the bedrooms, to ensure that patients can talk with HCPs via speakerphones from their whole apartment. Other patients express concerns about what will happen when their health status deteriorates and they become too sick to yell from their bedrooms, and the HCPs can’t hear them on the speakerphone. Furthermore, the experience of a device suddenly not working created confusion and fear. A patient described an episode of infection followed by confusion, when she was not rational and didn’t remember to activate the safety alarm. As she put it, “So *no point in a safety alarm then. Then you can count only on people*” (P3). Dependence increased, and the autonomy that wearable devices could offer was thus experienced as untrustworthy in situations when patients forget either how to use them, or log in to the application, or if body vulnerability makes them more dependent.

### Shifting Care Responsibilities Within the Context of Life’s Finitude

Wearing a device on the body creates a special bond between the wearer and the device, which can evoke mixed emotions. The participants expressed ambivalence: on the one hand, they treated their devices as treasures and felt responsible for them; on the other, they felt frustration due to technical issues or because the devices were seen as reminders of the finitude of their lives. Accordingly, the patients’ sense of dignity fluctuated, sometimes reflecting a sense of mutual care between themselves and their wearable devices, resulting in a bond they valued, and at other times dominated by poignant and sobering thoughts about the finitude of life potentiated by the devices’ limitations or dysfunctionalities.

A special bond was expressed through paying particular attention to and cherishing wearable devices, for instance, keeping safety alarms and pain pumps close by and protected when showering or taking notes on daily medication dosages dispensed through pain pumps. One patient said about her safety alarm: “It’s worth its weight in gold. I never take it off, not when showering, washing dishes, never” (P3). Wearable devices were experienced as a guarantee of safety, accepting responsibility for own health, as well as enabling help to be sought in critical situations or when simply wanting to get in touch with HCPs for a checkup. The constant wearing of pain pumps was compared to the regular carrying of a mobile phone.

Wearable devices as a regular part of care can also be a burden. In this example, the patient’s feeling of responsibility for the device caused him to be vigilant at night:

I can’t sleep because I must watch that the pain pump doesn’t disconnect and leave me in pain. So, I take care of the pump, and it takes care of me. I also expected it not to be so heavy to wear. (P5)

Even though patients are satisfied with the renewed ability to go to a restaurant, travel, or go for a walk instead of just lying in bed, for some, the device feels like a constant reminder of the finitude of life: “*Should I wear it till I die?*” Digitally monitored patients pointed out complicated and time-consuming processes of logging into applications connected to wearable devices. This process is required each time a patient enters the application, which is necessary if they want to take, record, or see their own measurements, fill in a questionnaire, chat, or call HCPs. Patients questioned the reliability of digital remote care because they were sometimes unable to see their own measurements, text, or call HCPs due to technical issues. Inability to remember login processes, or health deterioration, might hinder the login procedure, which altogether emphasises feelings of limitations and responsibility for one’s own health in these situations and potentiates their frailty and finitude. Patients followed by a digital monitoring department feel safe knowing that HCPs from the department will notice and respond if they fail to enter their daily measurements. Patients have varied attitudes to how much technology or wearable devices they can accept. These ranged from accepting as much as possible, wishing to have insight into own measurements and be in control, to accepting the bare minimum and refusing to take responsibility for own measurements. Even though patients do not question their privacy when using wearable devices, they draw a strict line against in-home cameras: “*We are old, but we still have our privacy*.”

### Uncertainty About Being Valued, Seen, and Empowered While Navigating Relational Expectations

Older patients in palliative care feel supported by HCPs and encouraged to use wearable devices without worrying about making mistakes with them. This creates a trusting relationship between them and a sense of being seen as a whole person, receiving holistic care. Patients, however, wonder if there is an unspoken limit to the care they can expect. They feel they should manage many things by themselves and be “good patients” rather than burdens. Contradictions in patients’ sense of dignity, such as feeling seen, valued, and empowered, yet aware of their vulnerability, put their sense of dignity at stake. A need to balance relational expectations to avoid becoming an encumbrance or making excessive demands is present.

HCPs working with wearable devices encourage patients to text, call, or activate safety alarms whenever they need help or an answer. They assure the patients they can’t make mistakes. This lays the foundation for patients’ feelings of safety, reliability, and trust in HCPs and wearable devices. For example, safety alarms can be activated by chance, but HCPs reassure patients that these are not treated as false alarms; instead, they are viewed as “try-outs.” Patients report feeling that they receive holistic care thanks to the mix of wearable devices and HCPs, in contrast to the example of visiting a cardiologist who looks at their heart and nothing else and doesn’t include them in the decision-making. Remote monitoring care gives a feeling of being seen not as anonymous patients but as whole persons, dignified, and in control. Patients insisted that *“care by using technology without HCPs would be cold. It might lead to separation and desocialisation from other people and feeling treated as a number. Without HCPs, care would not be humanised or dignified.”*

At the same time, out of respect for HCPs and a desire not to depend too much on others, the patients in this study try to do what they can on their own by giving themselves injections, ensuring an adequate medication supply, “being a good patient,” and avoiding unduly burdening the HCPs.

Older patients in palliative care in this study expected that all HCPs would have access to all patients’ health data. “*I gave them (HCPs) permission to send my measurements and all of my health data to everyone that needs them*.” (P1). The expectation includes sharing all their health information and cooperation as a team in treatment decisions, as well as including themselves. One patient explained that his family physician included him in the decision-making process regarding his treatment. He pointed out that specialists don’t do that, making him feel like a case number, rather than a person. In contrast, the family physician and the patient agreed on a revised medication list together, excluding some medications and adjusting doses. The patient felt supported, empowered, heard, and validated by this relationship. Contradictory lived experiences, like those described in the constituents of the phenomenon, exemplify a sense of dignity at stake.

## Discussion

The aim of the study was to gain insight into the phenomenon of how wearable devices affect the sense of dignity of older patients in palliative care settings using remote monitoring health services. The essence of the phenomenon that the sense of dignity is at stake for older adults over 65 in palliative care using wearable devices for remote health monitoring highlights the need to understand better what is at risk for these patients. Their experiences are marked by ambivalence, reflecting mixed and sometimes contradictory feelings about whether this type of care supports their dignity. In the Norwegian palliative care context, these older patients’ sense of dignity was deeply influenced by their perceptions of independence and dependence, which were closely tied to the progression of their illnesses and, in turn, impacted their sense of identity. The ambivalence in patients’ moods and attitudes towards wearable devices and remote home monitoring care might lead to ambiguity in HCPs’ understanding of patients’ sense of dignity. This, in turn, could challenge how HCPs acknowledged levels of dependency in the context of offering dignified care. According to the framework of Galvin and Todres’ (2015), dignity is a complex phenomenon, derived from lived perceptions that encompass all variations from vulnerability, respect, autonomy, honour, and kinship.

The essential structure of dignity as described by Galvin and Todres (2015) offers seven kinds of dignity: spatial, temporal, identity, interpersonal, mood, embodied, and finitude dignity. Awareness of these might help HCPs to recognise nuances in patients’ experiences. These kinds of dignity are intertwined and have variations in their ranges and intensities that can point to a rupture in or absence of dignity. They can also help us see when a sense of dignity is being honoured (Galvin & Todres, 2015). Furthermore, dignity is seen as both relational and experiential (Galvin & Todres, 2015). This renders it applicable for discussing the relational aspects of this study’s findings.

Our findings indicate that these patients believed that holistic and dignified palliative remote care cannot be provided without trustful relationships between patients and HCPs. Patients saw wearable devices as means to receive care and treatments from their homes (which supported spatial dignity), through contact with HCPs; they considered that they could rely on HCPs even when they had technical issues or felt limited by their bodies and illnesses. Encouragement, empowerment, and holistic care they got from HCPs are examples of supported interpersonal dignity. Interpersonal dignity is experienced in relationships characterised by mutual valuing expressed through safeguarding and respecting vulnerability (Galvin & Todres, 2015). Even though patients’ mood dignity ranged between feeling vulnerable and limited and free and independent, interpersonal dignity supported their mood dignity through mutual valuing and attentiveness. The phenomenon that the sense of dignity is at stake for older people in palliative care with wearable devices in remote monitoring health services highlights how different kinds of dignity are intertwined and dynamic, with varying intensities.

Interpersonal dignity requires HCPs to recognise nuances in patients’ understanding of dignified care. Viftrup et al. (2021) found differences in patients’ and HCPs’ experiences of dignity and dignified palliative care in hospices. HCPs considered that they provided dignity by allowing patients the autonomy to decide when the HCPs should interfere, while some patients from the same study felt abandoned and left alone in their hospice room. In contrast, HCPs in the study by Spaho et al. (2024) took more initiative for spending time with patients, forging the trusting relationships that factor decisively in providing individualised, dignified palliative care.

Other studies have underlined HCPs’ role in remote monitoring via wearable devices and telehealth, emphasising that HCPs can empower patients to be active partakers in their own health, enable feelings of safety, provide individual, patient-centred care, and early interventions in cases of changing health, prevent unnecessary readmissions through constant patient involvement (Hentschel et al., 2016; Kang & Exworthy, 2022; Vaismoradi et al., 2024). Improved communication via wearable devices and patient education makes the patients more active in decision-making, increasing their quality of life. (Kang & Exworthy, 2022) In contrast, the loss of human interaction that would result from using digital technology alone may lead to social isolation (Hentschel et al., 2016), especially among older people (Kang & Exworthy, 2022), and in palliative care, which itself carries a risk of alienation and isolation (Ott et al., 2023).

Temporal dignity is supported when the patients feel satisfied with their place in the world and time (Galvin & Todres, 2015). On the contrary, our patients believed their temporal dignity was denied when their voices were not considered when the institution decided to change night shift with safety alarms because of their age, fostering feelings of vulnerability, and questioning the appropriate level of care for their age and their value. This example illustrates how dismissing patients’ voices undermines not only their temporal, but also their mood and identity dignity, as well as the dignity of the older patients’ group identity. Inclusion of patients in the co-design of care interventions is a critical component for ensuring a personalised approach (Haase et al., 2020; LeBaron et al., 2022; Raja et al., 2023; Walton et al., 2022), but also a way of ensuring that their voices matter, and enabling support for dignified care.

Galvin and Todres (2015) define identity dignity as a person’s experience of meeting their own standards, incorporating body, time, space, and mood in their sense of self-value. Indignity is experienced when one cannot meet one’s standards (Galvin & Todres, 2015). Wearable devices supported patients’ identity dignity by enabling autonomy, deep self-identity, responsibility for own actions, being an active partaker in own health, and less dependent from others. Dignity was at stake when wearable devices caused feelings of diminished quality of life, making patients feel limited.

Wearable devices can directly affect patients’ embodied dignity, bodily “I can.” When a patient’s health status changes, the patient experiences corresponding changes in several kinds of dignity, including identity, embodiment, and finitude dignity. Bodily limitations and sudden health deteriorations (embodiment) act as reminders of the finitude of life for patients in palliative care and can make them more dependent on HCPs. However, this dependence was tacit, as patients were aware of these sudden health changes. Even though patients’ levels of independence and dependence were changing, their identity dignity was salvaged by experiencing empowerment and interpersonal dignity in their interactions with HCPs. Patients not only relied on but also expected help from HCPs, especially in these critical moments. This was reflected by their showing trust in HCPs and feeling supported, in control, and empowered. The use of wearable devices was causing feelings of pressure and responsibility in COVID-19 patients in cases of health deterioration (Walton et al., 2022). Some patients struggle with performing and reporting measurements and may lack the willingness to take on self-management. This emphasises the role of HCPs in assessing a patient’s readiness for this type of care (Kang & Exworthy, 2022; Vaismoradi et al., 2024).

Empowerment happens when persons believe in own abilities and in control to do tasks that reflect their personal values (Nelson et al., 2024; Zhang et al., 2024). Empowerment can be used to enhance adherence to digital device use, achieve desired health outcomes, and meet patients’ own goals (Nelson et al., 2024). For instance, Nelson et al. (2024) found a strong connection between acceptance of body-worn technology, or technology embodiment, with empowerment, which further enhanced user adherence to wearable device use. These findings suggest that HCPs can foster patients’ empowerment by instilling a sense of self-confidence that they can perform required tasks, while ensuring that they are in control. This includes offering choices and autonomy to make decisions that match patients’ own values.

Our findings, although based on a small sample, underline that patients want to be active participants in treatment decision-making, view their own measurements and results, and take responsibility for and control over their own lives. They believe that remote home monitoring care provides holistic care, in contrast to the narrow “regular” care, where they feel like anonymous patients. Although remote home care places responsibility on patients, they experience a supported sense of dignity both through the involvement of healthcare professionals and their own active participation in managing their health. Patients from other studies also emphasised the importance of having insight into their own symptom progression by using digital health technology, which enables them to feel empowered, in control, and secure in evaluating their own symptoms (Knotnerus et al., 2024; LeBaron et al., 2022).

Our findings demonstrate that dignity in remote monitoring by wearable devices is a multifaceted experience. By understanding the complexity of dignity as a phenomenon, healthcare professionals (HCPs) can be guided towards implementing practical interventions that promote meaningful and dignified care. This is particularly pertinent in palliative care settings.

## Strengths and Limitations

The strength of this study lies in its focus on the dignity at stake for this vulnerable group of older patients using wearable devices, while also raising important questions about individual and group identity dignity in palliative care. By deepening our understanding of how wearable devices influence the sense of dignity among older patients in remote monitoring settings, the study fosters greater awareness and supports the delivery of more dignified palliative care.

The report on the effects of the pilot implementation of digital monitoring departments in Norway was published in 2022 (Helsedirektoratet [Ministry of Health Department], 2022) while our recruiting was in progress. Since this health service was new in Norway, the overall pool of participants was limited, and the COVID-19 pandemic restrictions further restricted the number of participants who met the inclusion criteria of our study. Contextual factors should be considered when assessing the transferability of the findings. For instance, remote home monitoring services in Norway are provided free of charge, which may influence patients’ experiences and perceptions (Bodø kommune, n.d..; Helsedirektoratet [Ministry of Health Department], 2022). Furthermore, Norway is a welfare state with public health services that provide equal access and care to all citizens, regardless of their socioeconomic status or place of residence (Maagerø & Simonsen, 2021). Although remote home monitoring care services were in the project phase, and several cities (Helsedirektoratet [Ministry of Health Department], 2022), there are still discrepancies in the availability of remote home care using digital technology between the rural and urban areas in Scandinavian countries (Raja et al., 2023). Even though there are many wearable devices on the global market, from smartwatches and Fitbit activity trackers to devices that can monitor electrolyte levels or even smart wearable clothes, their adoption has been slow (Kang & Exworthy, 2022; Maguraushe & Ndlovu, 2024; Nelson et al., 2024). The range of devices available in this study was limited; however, it included patients using various types of wearable technology from safety alarms to home blood pressure monitors across different residential settings and models of remote palliative care. This diversity offered a range of patient perspectives, highlighting which technologies older patients in palliative care may be willing to accept and how. The devices used in our study had been in use for several years within Norwegian healthcare.

A strength of the study is that it includes the experiences of patients in this vulnerable group, all of whom were already receiving some form of remote monitoring care. Attrition is not uncommon in palliative care research, as seen in our study and others (Dunleavy et al., 2023; Froggatt et al., 2020; Oriani et al., 2020; Slev et al., 2020), with average rates starting around 29% (Oriani et al., 2020). Both qualitative and quantitative studies without our age limit, such as Wright et al. (2018) and Lowe et al. (2020), and those conducted in hospices (Froggatt et al., 2020), also reported high attrition rates and small sample sizes, yet made meaningful contributions to palliative care. Similarly, the modest sample size in our study enabled rich, in-depth exploration of lived experiences, aligning with phenomenological aims.

## Conclusion and Future Implications

This phenomenological study explored how wearable devices influence the sense of dignity among older patients receiving palliative care through remote home monitoring in Norway. The findings highlight that dignity is a multifaceted experience shaped by identity, mood, finitude, and interpersonal relationships and is deeply impacted by digital health technologies. These insights can help healthcare professionals deliver more individualised, patient-centred, and cost-effective remote palliative care. As remote care technologies evolve, recognising the possibilities and limitations for supporting dignity is essential. Further research with larger, more diverse samples is needed to expand understanding and inform actionable strategies. Stakeholders, including software developers, policymakers, care providers, and patients themselves, must collaborate to ensure remote home care systems protect and promote dignity in ageing and dying.
